# Approximate entropy detects the effect of a secondary cognitive task on postural control in healthy young adults: a methodological report

**DOI:** 10.1186/1743-0003-4-42

**Published:** 2007-10-30

**Authors:** James T Cavanaugh, Vicki S Mercer, Nicholas Stergiou

**Affiliations:** 1 Department of Physical Therapy, University of New England, Portland, ME, USA; 2 Department of Allied Health Sciences, The University of North Carolina at Chapel Hill, Chapel Hill, NC, USA; 3 HPER Biomechanics Laboratory, University of Nebraska at Omaha, Omaha, NE, USA

## Abstract

**Background:**

Biomechanical measures of postural stability, while generally useful in neuroscience and physical rehabilitation research, may be limited in their ability to detect more subtle influences of attention on postural control. Approximate entropy (ApEn), a regularity statistic from nonlinear dynamics, recently has demonstrated relatively good measurement precision and shown promise for detecting subtle change in postural control after cerebral concussion. Our purpose was to further explore the responsiveness of ApEn by using it to evaluate the immediate, short-term effect of secondary cognitive task performance on postural control in healthy, young adults.

**Methods:**

Thirty healthy, young adults performed a modified version of the Sensory Organization Test featuring single (posture only) and dual (posture plus cognitive) task trials. ApEn values, root mean square (RMS) displacement, and equilibrium scores (ES) were calculated from anterior-posterior (AP) and medial-lateral (ML) center of pressure (COP) component time series. For each sensory condition, we compared the ability of the postural control parameters to detect an effect of cognitive task performance.

**Results:**

COP AP time series generally became more random (higher ApEn value) during dual task performance, resulting in a main effect of cognitive task (p = 0.004). In contrast, there was no significant effect of cognitive task for ApEn values of COP ML time series, RMS displacement (AP or ML) or ES.

**Conclusion:**

During dual task performance, ApEn revealed a change in the randomness of COP oscillations that occurred in a variety of sensory conditions, independent of changes in the amplitude of COP oscillations. The finding expands current support for the potential of ApEn to detect subtle changes in postural control. Implications for future studies of attention in neuroscience and physical rehabilitation are discussed.

## Introduction

Over the last two decades, dual task studies examining the role of attention in postural control have become increasingly important in clinical neuroscience [1,2], engineering [3], and physical rehabilitation [4,5]. However, while techniques for evaluating attention have become more sophisticated [6], dual task methods for evaluating postural control have progressed little. Specifically, researchers generally have relied upon degraded postural stability, operationally defined as an increase in the amplitude of center of pressure (COP) variability, to indicate interference from a secondary cognitive task [7-15]. Postural stability measures, perhaps because of their relatively limited precision [16], have not consistently revealed changes in postural control during cognitive perturbations [7,10,12,15,17]. Thus, the automaticity of postural control remains the subject of ongoing debate [18].

As an alternative measure of postural control, approximate entropy (ApEn) has been used to quantify COP variability during quiet standing [19]. ApEn quantifies the amount of irregularity, or randomness, in a time series [20]. A small but growing body of evidence supports the use of ApEn for detecting subtle changes in COP variability that are not necessarily apparent using biomechanical measures of postural stability [21,22]. Moreover, ApEn has demonstrated relatively high response stability and precision for repeated trials of quiet standing within a single session [16]. This particular quality suggests that ApEn might be useful in dual task studies of attention, in which changes in postural control typically are evaluated over very short time intervals.

Our purpose in the present study was to use a dual task paradigm to conduct a preliminary evaluation of whether ApEn could detect a short-term change in postural control in response to the addition of a secondary cognitive task. To minimize the influence of age and pathology, we selected healthy, young adults as participants. We used a modified version of a common clinical test battery, the Sensory Organization Test (SOT; NeuroCom, Inc., Clackamas, OR), to test dual task performance under various sensory conditions. In contrast to a motoric challenge, the SOT provided an opportunity to collect steady-state (i.e., quiet standing) postural control data suitable for the application of ApEn. To further understand the potential utility of ApEn, we compared its ability to detect subtle change with that of two biomechanical measures of COP amplitude (root mean square (RMS) displacement and the SOT Equilibrium Score (ES)). We hypothesized that ApEn would be more likely than RMS values and ES to detect a change in postural control associated with the performance of a secondary cognitive task.

## Methods

### Subjects

Thirty students (15 males and 15 females; mean age = 21.7, SD = 2.3 yrs; mean weight = 71.0, SD = 13.3 kg; mean height = 173.0, SD = 11.0 cm) from the University of North Carolina at Chapel Hill (UNC-CH) volunteered to participate. Subjects reported no history of neurological or musculoskeletal pathology that might affect postural steadiness. All subjects were non-smokers and denied ingesting within 24 hours prior to testing any substance (dietary, pharmacological, or recreational drug) that might affect motor performance. To avoid potential physiologic confounders, subjects were required to avoid vigorous physical activity within 2 hours of testing and to be free of pain, dizziness, or unusual fatigue. Subjects were paid for their participation and signed an informed consent form approved by the UNC-CH Institutional Review Board.

### Instrumentation

**Figure 1:**
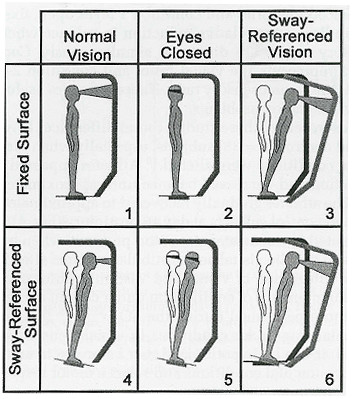
Sensory Organization Test (SOT)-Six Conditions. Used courtesy NeuroCom International, Inc.

The SOT was conducted in a quiet room using a Smart Balance Master System 8.0 (NeuroCom International, Inc., Clackamas, OR, USA), a widely accepted clinical instrument that has been used to detect abnormal postural control and to monitor the recovery of postural control after injury [23-28]. The system was equipped with a moveable visual surround and support surface that could rotate in the AP plane. Two 22.9 × 45.7 cm force plates connected by a pin joint were used to collect COP coordinates at 100 Hz. Subjects were instructed to stand still with their arms relaxed at their sides and while looking straight ahead, without reaching out to touch the visual surround or taking a step. Subjects wore comfortable attire, including socks, but were shoeless during testing. Foot placement was standardized based on subject height according to manufacturer guidelines. A safety harness secured overhead was used to prevent falling to the floor. The SOT systematically manipulates various combinations of visual, vestibular, and somatosensory stimulation in six sensory conditions (Figure 1).

The ability to stand as still as possible was evaluated under single task (standing still) and dual task (standing still plus digit recall) modes [29]. To normalize the challenge of the digit recall task, we first determined each subject's unique digit span by having the examiner (JTC) recite a four-digit, random number string aloud at a slow, deliberate pace. Subjects, while seated, were asked to repeat the string as accurately as possible. With each correct response, the examiner recited a new number series, one digit longer than the previous string. If a string was recalled inaccurately, a second attempt was offered at the same length using a new set of digits. Digit span was defined as the maximum string length that could be recalled accurately.

We modified the traditional SOT protocol as follows. After brief practice, subjects performed one single task trial and one dual task trial in random order for each sensory condition. Sensory conditions were presented in ascending order. For dual task trials, the examiner began by reciting a string of random numbers, equal in length to the subject's predetermined (seated) digit span. Upon reciting the final digit in the string, the examiner initiated COP recording, and the subject repeated the digit string aloud as accurately as possible while trying to stand still. Recitation and repetition of new random number strings, all of equal length, continued without hesitation until the 20-second SOT trial had been completed.

### Data reduction and analysis

#### ApEn

Generally speaking, the ApEn algorithm quantifies randomness by determining the extent to which short sequences of data points are repeated in a time series. More precisely, the ApEn algorithm calculates the logarithmic probability that runs of patterns that are close (i.e., within error tolerance *r*) for *m *observations remain close on subsequent incremental comparisons. To calculate ApEn for a time series containing *N *data points, *u(1), u(2), ..., u(N)*, an operator inputs (1) *m*, a pattern length, and (2) *r*, an error tolerance. The first step is to form vector sequences *x(1) *through *x(N - m - 1) *from the *{u(i)}*, defined by *x(i) = [u(i), ..., u(i + m - 1)]*. These vectors are basically *m *consecutive *u *values, beginning with the *i-th *point. The second step is to define the distance *d [x(i),x(j)] *between vectors *x(i) *and *x(j) *as the largest difference in their respective scalar components. The third step is to use the vector sequences *x(1) *through *x(N - m - 1) *to create (for each *i ≤ N - m + 1*)

Cim(r)=(number of x(j) suchthat d[x(i),x(j)]≤r)/(N−m+1)
 MathType@MTEF@5@5@+=feaafiart1ev1aaatCvAUfKttLearuWrP9MDH5MBPbIqV92AaeXatLxBI9gBaebbnrfifHhDYfgasaacH8akY=wiFfYdH8Gipec8Eeeu0xXdbba9frFj0=OqFfea0dXdd9vqai=hGuQ8kuc9pgc9s8qqaq=dirpe0xb9q8qiLsFr0=vr0=vr0dc8meaabaqaciaacaGaaeqabaqabeGadaaakeaacqWGdbWqdaqhaaWcbaGaemyAaKgabaGaemyBa0gaaOGaeiikaGIaemOCaiNaeiykaKIaeyypa0JaeiikaGIaeeOBa4MaeeyDauNaeeyBa0MaeeOyaiMaeeyzauMaeeOCaiNaeeiiaaIaee4Ba8MaeeOzayMaeeiiaaIaeeiEaGNaeiikaGIaeeOAaOMaeiykaKIaeeiiaaIaee4CamNaeeyDauNaee4yamMaeeiAaGMaeeiDaqNaeeiAaGMaeeyyaeMaeeiDaqNaeeiiaaIaeeizaqMaei4waSLaeeiEaGNaeiikaGIaeeyAaKMaeiykaKIaeiilaWIaeeiEaGNaeiikaGIaeeOAaOMaeiykaKIaeiyxa0LaeyizImQaemOCaiNaeiykaKIaei4la8IaeiikaGIaemOta4KaeyOeI0IaemyBa0Maey4kaSIaeGymaeJaeiykaKcaaa@6CD1@

The Cim(r)
 MathType@MTEF@5@5@+=feaafiart1ev1aaatCvAUfKttLearuWrP9MDH5MBPbIqV92AaeXatLxBI9gBaebbnrfifHhDYfgasaacH8akY=wiFfYdH8Gipec8Eeeu0xXdbba9frFj0=OqFfea0dXdd9vqai=hGuQ8kuc9pgc9s8qqaq=dirpe0xb9q8qiLsFr0=vr0=vr0dc8meaabaqaciaacaGaaeqabaqabeGadaaakeaacqWGdbWqdaqhaaWcbaGaemyAaKgabaGaemyBa0gaaOGaeiikaGIaemOCaiNaeiykaKcaaa@33CF@ values measure (within the tolerance *r*) the regularity of patterns similar to a given pattern of window length *m*. The fourth step is to define Φ^*m*^(*r*) as the average value of ln Cim(r)
 MathType@MTEF@5@5@+=feaafiart1ev1aaatCvAUfKttLearuWrP9MDH5MBPbIqV92AaeXatLxBI9gBaebbnrfifHhDYfgasaacH8akY=wiFfYdH8Gipec8Eeeu0xXdbba9frFj0=OqFfea0dXdd9vqai=hGuQ8kuc9pgc9s8qqaq=dirpe0xb9q8qiLsFr0=vr0=vr0dc8meaabaqaciaacaGaaeqabaqabeGadaaakeaacqWGdbWqdaqhaaWcbaGaemyAaKgabaGaemyBa0gaaOGaeiikaGIaemOCaiNaeiykaKcaaa@33CF@, where ln is the natural logarithm. Lastly, we define Approximate Entropy as

ApEn*(m,r,N) *= Φ^*m*^(*r*) - Φ^*m*+1 ^(*r*)

ApEn generates a unit-less real number from 0 to 2 [30]. Smaller ApEn values indicate a higher probability of regularly repeating sequences of *m *observations. An ApEn value of zero, for example, corresponds to a time series that is perfectly repeatable (i.e., sine wave). An ApEn value of 2 is produced by random time series, for which any repeating sequences of points occur by chance alone (i.e., Gaussian noise).

Using Matlab software (Mathworks, Natick, MA), we calculated separate ApEn values for the AP and ML components of the COP coordinate time series (N = 2000) from each test trial. Input parameters for the ApEn calculation were (1) a pattern length (*m*) of 2 data points, (2) a tolerance window (*r*) normalized to 0.2 times the standard deviation of individual time series, and (3) a lag value of 10. The pattern length (m) and tolerance value (r) were selected based on previous work [21,31-33]. The lag value of 10 dictated that the ApEn calculation include every 10^th ^point the raw time series. We chose this lag value to lower the effective sampling frequency of the algorithm from 100 Hz to 10 Hz, thereby reducing the influence of extraneous noise in the data.

As a necessary component of nonlinear dynamics methodology, we also applied a surrogation (phase randomization) procedure to verify that COP data were derived from a deterministic source [34]. Surrogate AP and ML time series were created having identical means, standard deviations, and power spectra to the original data but with randomly generated order. This procedure also was performed in Matlab using the algorithms developed by Theiler et al [34-36]. ApEn values from the original data and their surrogated counterparts were compared using the Student t-test (α = .05). The procedure revealed that ApEn values for the original time series were significantly less than for their respective surrogated counterparts, indicating that the original data were not randomly derived, and therefore, were deterministic in nature.

#### RMS displacement

RMS denotes the average spread of a time series distribution relative to its mean. For our purpose, RMS was calculated for each test trial as the square root of the mean squared deviation from the average COP value. Separate RMS values were calculated for the COP AP and ML time series components (N = 2000). Higher RMS values indicate greater variability, traditionally interpreted as greater postural instability. RMS values have been previously used in dual task studies of postural control in healthy, younger adult samples [11,17].

#### Equilibrium score

An ES was generated for each trial based on an algorithm developed for the SOT [37]. The algorithm uses the peak-to-peak amplitude (range) of COP AP displacement to estimate the amount of postural sway in the AP plane. Scores are calculated as the angular difference, expressed as a percentage, between the amount of estimated AP postural sway and the theoretical limit of stability, approximately 12.5° in the AP plane [37]. Lower amplitudes of postural sway require less COP displacement to control and produce higher percentage differences from the theoretical limit. Thus, a higher ES indicates greater postural stability in the AP plane. No analogous ES exists for the COP ML component. Although similar in construct to RMS values, we chose to analyze ES because of its common clinical use in conjunction with the SOT. Like RMS, ES values also have been previously used in dual task research [10].

All statistical analyses were conducted using SPSS 11.0 software (SPSS, Inc., Chicago, IL). We applied separate 2 × 6 (cognitive task × sensory condition) repeated measures analyses of variance (ANOVA) for ApEn values, RMS displacement and ES (α = 0.05) generated from single and dual task trials. Due to violations of Mauchly's sphericity assumption, we adjusted the ANOVA results using the more conservative Geisser-Greenhouse F-test.

## Results

No significant interaction was found between cognitive task and sensory condition for ApEn-AP and ApEn-ML values (Table 1). COP AP time series became more random (higher ApEn value) during dual task performance, resulting in a main effect for the cognitive task [F(1,29) = 9.93, p = 0.004]. In contrast, there was no significant effect of cognitive task for ApEn-ML values [F(1,29) = 0.94, p = 0.34]. Neither RMS displacement nor ES revealed a significant interaction between cognitive task and sensory condition or a main effect of cognitive task.

**Table 1:** Mean (standard deviation) parameter values during single and dual task conditions.

Parameter	SOT	AP		ML	
		Single	Dual	Single	Dual

ApEn	1	.810 (.15)	.819 (.17)	1.006 (.21)	.902 (.30)
	2	.753 (.15)	.791 (.16)	.902 (.26)	.947 (.22)
	3	.673 (.15)	.770 (.19)	1.020 (.23)	.990 (.32)
	4	.567 (.27)	.613 (.22)	.926 (.19)	.861 (.24)
	5	.604 (.16)	.649 (.19)	.810 (.21)	.874 (.15)
	6	.474 (.16)	.548 (.22)	.849 (.16)	.837 (.19)
RMS	1	.222 (.06)	.223 (.07)	.104 (.04)	.127 (.09)
	2	.384 (.13)	.370 (.11)	.152 (.11)	.132 (.07)
	3	.400 (.12)	.395 (.13)	.105 (.04)	.120 (.07)
	4	1.040 (.92)	.994 (.85)	.151 (.09)	.172 (.10)
	5	1.749 (.69)	1.713 (.89)	.287 (.14)	.235 (.09)
	6	2.164 (.87)	2.073 (1.2)	.212 (.09)	.227 (.11)
ES	1	95.6 (1.3)	95.7 (1.5)	n/a	n/a
	2	92.9 (2.3)	92.8 (2.5)	n/a	n/a
	3	92.1 (2.8)	93.7 (2.2)	n/a	n/a
	4	83.7 (12.2)	83.3 (12.4)	n/a	n/a
	5	68.8 (11.6)	70.3 (14.0)	n/a	n/a
	6	65.3 (12.0)	65.9 (16.1)	n/a	n/a

Approximate Entropy (ApEn) Values, Root Mean Square (RMS) displacement values, and Equilibrium Scores (ES) were based on center of pressure (COP) time series produced during six Sensory Organization Test (SOT) sensory conditions. Higher ApEn values indicate greater COP randomness (less system constraint). Higher RMS values and lower ES indicate greater COP amplitude (greater postural instability). n/a: not applicable; ES are calculated only from the COP AP component. SOT conditions are defined in Figure 1.

All subjects completed the SOT without taking a step or using hand support to maintain control of upright standing. Subjects' digit spans ranged in length from 5 to 10 digits (mean 7.2 ± 1.2). Twenty-six subjects (86.7 %) completed two digit strings for each dual task trial, while four subjects (13.3 %) completed three digit strings. Twenty-two subjects (73%) made digit recall errors in at least one string during conditions 1 and 5, twenty subjects (67%) made errors in conditions 2 through 4, and fifteen subjects (50%) made errors in condition 6. The relatively high frequency of digit recall errors indicated that the cognitive task was burdensome enough to potentially interfere with postural control.

For every test trial, mean ApEn values from the surrogate AP and ML time series were significantly larger than their original counterparts (all probability values were less than 0.01), indicating that the original COP data were deterministic, rather than randomly derived. This result justified the application of nonlinear methods to the analysis of COP time series [32].

## Discussion

During performance of a secondary cognitive task, ApEn detected a change in COP variability that was not detected by RMS or ES. We believe that this finding primarily results from differences in underlying measurement construct. ApEn, as a highly iterative procedure, considers the sequential order of neighboring data points in a COP time series. RMS values and ES, however, reflect the overall magnitude of COP displacement, without consideration of temporal order. This fundamental difference may explain why nonlinear algorithms often reveal subtle time series properties not detected previously using the traditional linear approach [19,21,22]. The distinction may also explain why ApEn values, in particular for COP AP time series, have demonstrated relatively higher measurement precision in comparison to RMS and ES when applied to COP time series recorded from healthy, young adults [16]. Higher precision inherently implies greater measurement responsiveness.

Another possible explanation for our findings is that performance of the secondary cognitive task produced a change in the allocation of attention that uniquely affected ApEn values. How such reallocation is thought to occur remains a matter for theoretical debate [18,38,39]. According to a "facilitory-control" view [38], the increased randomness in COP oscillations may have occurred in an effort to facilitate the supra-postural cortical task of recalling digits aloud, presumably via a shift in attentional resources. A different interpretation would suggest that the instruction to "stand as still as possible" during the posture-only task placed a somewhat unusual (novel) constraint on what commonly is a well learned yet unrestricted task (standing quietly). By focusing attention on the task of standing still, subjects may have artificially constrained the interactions among underlying postural control system components, thereby increasing the regularity of the output signal. A similar suggestion has been proposed elsewhere [14,40] and requires further investigation.

Alternatively, one might speculate according to a classic "autonomous-control" view of postural control [41] that changes in COP regularity were produced not by a reallocation of attention but by mechanical destabilization, albeit along a temporal rather than a spatial dimension, brought about by articulation and respiratory patterns during the spoken cognitive task. Previous studies have shown, for example, that mechanical effects from articulation and respiration during dual task performance influence the amplitude of COP variability even in the absence of a changing attentional demand [9,11]. Whether the mechanical influence of vocalization extends to the temporal structure of COP variability remains unclear.

Were the changes in ApEn during dual task performance large enough to be clinically important? We acknowledge that even the largest mean ApEn changes (Conditions 3 and 6) were only equivalent to approximately one standard error of measurement [16]. Nonetheless, we believe that our data indicate that ApEn shows promise for detecting subtle change in postural control independent of traditional biomechanical measures, even in a relatively small sample. More research is needed to confirm the current findings, expand our understanding of what constitutes meaningful clinical change in ApEn values, and determine the sensitivity and specificity of ApEn for detecting differences among diagnostic groups.

### Implications for future research

Practical measures that detect subtle changes in postural control are potentially important for advancing current understanding of attention and have broad implications for clinical neuroscience and physical rehabilitation. The present study suggests that traditional biomechanical measures of postural stability, which have dominated the dual task attention literature for two decades, should not necessarily be relied upon as the sole means of detecting subtle change in postural control. Indeed our findings indicate that a change in the temporal structure of COP variability appears to occur in response to the performance of a secondary cognitive task, independent of changes in postural stability. Regardless of the proposed underlying mechanism for this change, the direct implication of this finding is that future dual task studies of attention and postural control may be enhanced through the application of multiple postural control measurement frameworks.

Implementation of ApEn in postural control research undoubtedly will require more rigorous validation. Our work was preliminary; we made several methodological choices that highlight the need for confirmatory studies. Specifically, (1) we chose pattern length (m) and error tolerance (r) values based on previous studies but did not explore the potential impact of using alternative values; (2) we selected a lag value of 10 for the ApEn calculation (i.e., we lowered the effective sampling frequency to lessen the influence of extraneous noise) but did not similarly shorten the COP time series for the RMS and ES calculations; (3) we elected not to randomize the presentation of sensory conditions across subjects in an effort to mimic what we believe is common clinical practice with the SOT. This strategy eliminated the opportunity to analyze the effect of sensory condition (although the interested reader is invited to consult our previous analyses of this effect [21,31].)

An important implication of our study is that theoretical models describing the interplay between attention and postural control, even in more recent articulations [42], may require careful reexamination. Although our study is preliminary, the data suggest that during the simultaneous performance of a well-learned, non-demanding postural task (e.g., quiet, unperturbed standing with feet apart) and an attention demanding cognitive task (e.g., digit recall), healthy, young adults generate COP oscillations that are not only low in amplitude but also are relatively random compared to quiet standing alone. Said differently, automatic postural control in quiet standing (i.e., postural control that requires few attentional resources to maintain stability) may be characterized by high precision *and*relatively low constraint. In this context, "constraint" is operationally defined by the temporal structure (i.e., randomness) of COP oscillations. Nonlinear measures like ApEn are useful as indices of relative constraint, because in theoretical terms they are interpreted as a characterization of the dynamic interactions among components within the underlying control system [43]. More constrained postural control systems hypothetically produce lower ApEn values, whereas less constrained systems produce higher ApEn values [19]. Thus, we believe that rather than viewing attention as a stabilizing vs. destabilizing influence on postural control, perhaps a more informative framework would be to view attention as one of many constraints on postural task performance [44]. ApEn, therefore, may prove useful in future studies of attention as a reliable and responsive indicator of global postural control system constraint.

At the very least, our findings support the continued exploration of ApEn as a tool for detecting subtle change in COP variability not typically detected by traditional biomechanical measures. Indeed, measures like ApEn might be useful in a variety of other clinical applications. In physical rehabilitation, patients whose postural stability does not improve with intervention could be evaluated using ApEn to determine the nature of any neurophysiologic constraints that might be limiting improvement [40]. In sports medicine, athletes with minor musculoskeletal or neuromuscular injury who appear to have normal balance (using clinical measures of postural stability) might be evaluated using ApEn in an attempt to determine readiness to resume competition [21,31]. In pharmacology research, ApEn might be used to identify subtle effects of medication on postural control, which could have important implications especially for older adults at risk for falls [45]. Together these examples highlight the importance of efforts to generate alternative models of movement variability [46] that serve to improve the array of measurement alternatives available for postural control research.

## Conclusion

ApEn, as a measure for characterizing the temporal dynamics of COP variability, shows promise for detecting the immediate, short-term effect of secondary cognitive task performance on postural control during quiet standing, even among healthy subjects whose postural sway in this position is minimal. Our results highlight differences between the linear and nonlinear measurement approaches and supports their combined use in clinical neuroscience and physical rehabilitation research.

## Competing interests

The author(s) declare that they have no competing interests.

## Authors' contributions

JTC developed the study concept and design, collected study data, completed the data analysis and interpretation, and prepared the manuscript. VSM and NS participated in the development of the study concept and design, data interpretation, and manuscript preparation. All authors read and approved the final manuscript.
